# Safety and Efficacy of TEE Guidance in Electrophysiological Procedures Without Fluoroscopy

**DOI:** 10.3390/jcm14061917

**Published:** 2025-03-12

**Authors:** Lyuboslav Katov, Theresa Kistner, Yannick Teumer, Federica Diofano, Carlo Bothner, Wolfgang Rottbauer, Karolina Weinmann-Emhardt

**Affiliations:** Department of Cardiology, Ulm University Heart Center, Albert-Einstein-Allee 23, 89081 Ulm, Germanytheresa.kistner@uniklinik-ulm.de (T.K.); yannick.teumer@uniklinik-ulm.de (Y.T.); federica.diofano@uniklinik-ulm.de (F.D.); carlo.bothner@uniklinik-ulm.de (C.B.); wolfgang.rottbauer@uniklinik-ulm.de (W.R.)

**Keywords:** zero fluoroscopy, near-zero fluoroscopy, left atrial electrophysiological procedures, 3D mapping, EAM, transesophageal echocardiography, radiation exposure

## Abstract

**Background/Objectives**: Fluoroscopy has traditionally supported three-dimensional (3D) electroanatomical mapping (EAM)-guided left atrial (LA) electrophysiological procedures (EPs), but the associated ionizing radiation poses long-term health risks for patients and healthcare professionals. Advances in 3D EAM systems now enable nearly or entirely radiation-free ablations. Imaging techniques such as transesophageal echocardiography (TEE) are increasingly used for precise and safe LA access. This study evaluates the safety and efficacy of TEE-guided, zero-fluoroscopy/near-zero-fluoroscopy LA EPs in routine clinical practice. **Methods**: 142 consecutive patients undergoing LA EAM-guided radiofrequency ablation at the Ulm University Heart Center between October 2023 and November 2024 were analyzed. In total, 73 patients underwent zero-fluoroscopy/near-zero-fluoroscopy ablation guided solely by TEE, while another 69 patients received fluoroscopy-guided ablation using TEE and fluoroscopy guidance. **Results**: Of the 142 patients, 58.0 (40.8%) were female, and the median age was 73.0 (64.0; 79.0) years. A total of 53 (37.3%) underwent zero-fluoroscopy EP, 20 (14.1%) underwent near-zero-fluoroscopy EP, and 69 (48.6%) underwent fluoroscopy-guided EP. Procedure duration was without significantly relevant difference between both groups (132.0 vs. 133.0 min; *p* = 0.52). Median radiation exposure in the zero-fluoroscopy/near-zero-fluoroscopy group was 0 (0.0; 0.0) minutes, compared to significantly higher values in the fluoroscopy group (9.7 (5.9; 15.3) minutes; *p* < 0.001). No significant differences in complications were observed (*p* = 0.09). **Conclusions**: TEE-guided, radiation-free LA EP offers a safe and effective approach, significantly reducing radiation exposure and its associated risks while maintaining high procedural efficacy without increasing the risk of complications.

## 1. Introduction

Complex atrial arrhythmias such as atrial fibrillation (AF) and atypical atrial flutter (AAFL) predominantly originate in the left atrium (LA). Catheter ablation (CA) has emerged as a cornerstone treatment for these arrhythmias, particularly with the advancements in imaging and mapping technologies that enhance procedural safety and precision while minimizing radiation exposure [1].

Historically, fluoroscopy has been the primary imaging method during CA procedures, providing real-time X-ray guidance. However, the associated ionizing radiation poses significant risks, including deterministic (e.g., skin injuries and cataracts [2,3]) and stochastic (e.g., long-term risks, such as cancer) effects [4]. Importantly, there is no clearly defined threshold for safe radiation exposure [5], meaning that even low levels may carry cumulative risks over a lifetime for both patients and healthcare staff [6].

In interventional electrophysiology, radiation exposure varies by procedure complexity. Simple diagnostic electrophysiology studies typically result in an average dose of 3.2 mSv, while more complex procedures, such as atrial fibrillation ablation, have a mean effective dose of up to 20.3 mSv [7]. Recent advancements such as three-dimensional (3D) electroanatomic mapping (EAM) systems have significantly transformed electrophysiology procedures (EPs). These systems offer real-time, detailed visualization of cardiac anatomy and electrical activity, enabling greater precision in ablation procedures while substantially reducing or even eliminating the need for fluoroscopy [8]. This innovation minimizes radiation exposure, improving safety for both patients and medical personnel. However, radiation cannot always be avoided due to complex cardiac anatomy or the presence of intracardiac leads. In such cases, a near-zero-fluoroscopy approach has been pursued, with a fluoroscopy time of less than 1–2 min or a dose below 50–100 cGy·cm^2^ [9,10].

Different imaging modalities are increasingly employed to ensure precise and safe navigation during EP, especially in achieving LA access. Techniques such as intracardiac echocardiography (ICE) and the integration of computed tomography or magnetic resonance imaging are gaining importance in this field. Recent studies have highlighted the benefits of ICE guidance, enabling radiation-reduced or even completely radiation-free ablations [11,12,13,14]. Moreover, Chen et al. presented data showing the safe performance of transseptal punctures using only a unipolar electrogram at the needle tip in conjunction with 3D EAM [15]. The feasibility of transesophageal echocardiography (TEE) guidance in the treatment of structural cardiac interventions, as well as in electrophysiological procedures, e.g., during pulmonary vein isolation (PVI), has been previously demonstrated [16,17]. However, limited data exist on the role of TEE guidance in zero-fluoroscopy LA EP, which is why the current study aims to evaluate its safety and efficacy in real-time clinical practice.

## 2. Materials and Methods

### 2.1. Study Population

A total of 142 consecutive patients who underwent left atrial EP using 3D EAM and radiofrequency (RF) ablation were prospectively enrolled at the Ulm University Heart Center between October 2023 and November 2024. The initial cohort of 69 patients received treatment with fluoroscopic and TEE guidance (fluoro group), while the subsequent group of 73 patients underwent zero-fluoroscopy/near-zero-fluoroscopy procedures with the aid only of TEE guidance (zero-fluoro/near-zero-fluoro group). Both first-time and redo procedures were included. Patients under the age of 18, those requiring right atrial or left ventricular ablation, and individuals with contraindications to TEE were excluded from the study. Written informed consent was obtained from all participants before the procedures. The study received approval from the ethics committee of the University of Ulm and was conducted in accordance with the principles set forth in the Declaration of Helsinki. Data were prospectively collected as part of the ATRIUM registry (German Clinical Trials Register ID: DRKS00013013).

### 2.2. Periprocedural Management

The patients remained on continuous oral anticoagulation therapy without interruption. Our standard sedation protocol includes an initial bolus of 5 mg midazolam, followed by continuous, individually titrated administration of propofol (e.g., 2–6 mg/kg/h) for deep sedation and remifentanil (e.g., 0.05–0.2 µg/kg/min) for analgesia [18]. Following sedation, a TEE probe (Philips CX50 ultrasound system with a Philips X7 TEE probe, Philips, Amsterdam, The Netherlands) was inserted to rule out the presence of atrial thrombus and to provide further ultrasound guidance. If required, an esophageal multi-electrode temperature probe (S-Cath, Circa Scientific LLC, Englewood, CO, USA) was introduced transnasally for esophageal temperature monitoring during the ablation of the LA posterior wall. In the zero-fluoro/near-zero-fluoro group, a 3D mapping-integrated temperature probe was utilized.

### 2.3. Zero-Fluoroscopy/Near-Zero-Fluoroscopy Procedure Steps (Zero-Fluro/Near-Zero-Fluoro Group)

For the 3D EAM procedures, 3 to 4 venous punctures were performed under ultrasound guidance in the right groin [19]. Based on the mapping system used in the study, an 3D EAM of the right atrium (RA) was created. With two of the three available systems in our EP lab (Carto™, Johnson & Johnson, New Brunswick, NJ, USA; Rhythmia™, Boston Scientific, Marlborough, MA, USA), the 3D EAM of the RA was created using the corresponding high-density (HD) mapping catheter (Octaray™ and Pentaray™, Johnson & Johnson, New Brunswick, NJ, USA, or Orion™, Boston Scientific, Marlborough, MA, USA, respectively). The third system, EnSite X™ (Abbott, Chicago, IL, USA), utilizing NavX Mode, uniquely enables real-time visualization of each catheter immediately upon femoral access. Following this, a steerable 10-pole catheter (Inquiry™ Steerable Diagnostic Catheter, Abbott, North Chicago, IL, USA) was introduced into the coronary sinus (CS). The placement of the CS catheter into the RA through 7F groin sheath, as well as that of the aforementioned mapping catheters through either 8F or 9F groin sheath, was performed using smooth, resistance-free movements. If resistance was encountered, the catheter was retracted, rotated, and advanced again carefully. Upon reaching the RA and confirming the position via TEE, precise visualization in the 3D EAM system facilitated accurate catheter placement at the desired location.

**Figure 1:**
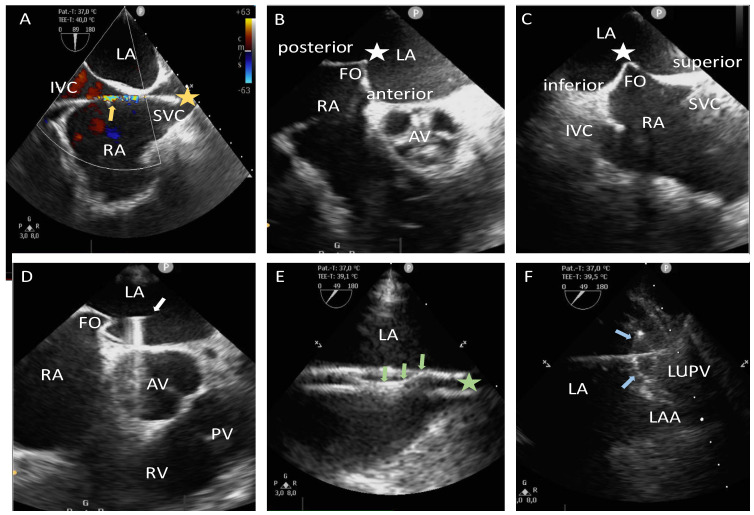
Illustration of performing TSP under TEE guidance. The upper left image (**A**) shows a bicaval view highlighting the passage of a 0.035-inch J-tip guidewire (yellow star) from the IVC through the RA into the SVC, along with typical color Doppler artifacts of the guidewire (yellow arrow). The upper middle image (**B**) depicts the mid positioning of the TSP system at the FO (white star) in the mid-esophageal short-axis TEE view. Correspondingly, the upper right image (**C**) shows the mid position of the TSP system at the FO (white star) in the bicaval view. The lower left image (**D**) illustrates the introduction of the coronary guidewire (white arrow) through the lumen of the TSP needle into the LA, subsequently advancing into the LUPV. The lower middle image (**E**) demonstrates the retraction of the guidewire (green arrows) following sheath placement into the LUPV (green star). The lower right image (**F**) shows the positioning of the mapping catheter (blue arrows) into the ostium of the LUPV. AV, aortic valve; FO, fossa ovalis; IVC, inferior vena cava; LAA, left atrial appendage; LA, left atrium; LUPV, left upper pulmonary vein; PV, pulmonary valve; RA, right atrium; RV, right ventricle; SVC, superior vena cava; TEE, transesophageal echocardiography; TSP, transseptal puncture.

The next step is crucial for the zero-fluoroscopy procedure. It is essential to accurately visualize the 0.035-inch J-tip guidewire in the superior vena cava (SVC) using TEE in the bicaval view, typically at a 90–110-degree imaging angle (Figure 1A). Under ultrasound guidance, the non-steerable sheath (CardiaGuide™, Johnson & Johnson, New Brunswick, NJ, USA) was advanced over the J-tip guidewire into the SVC. After removing the dilator and the J-tip guidewire, the double contour of the non-steerable sheath was observed. The transseptal puncture (TSP) needle (HeartSpan™ Transseptal Needle™, Johnson & Johnson, New Brunswick, NJ, USA) with integrated pressure measurement system was then introduced through the non-steerable sheath’s lumen until the double contour disappeared from the TEE view, indicating correct placement. It is crucial to maintain a distance of approximately 1–2 cm between the tip of the non-steerable sheath and the TSP needle to avoid unintended injury to the SVC. Subsequently, under TEE guidance in the bicaval view, the TSP system was retracted until reaching the fossa ovalis in the mid position. Afterward, the anteroposterior orientation of the TSP system was adjusted using the short-axis TEE view at 45 degrees (Figure 1B,C). For our purposes, a mid position was also preferred [20]. If the intended site was not reached, the TSP needle was withdrawn, and the procedure was restarted from the beginning with the placement of the non-steerable catheter in the SVC. Once the optimal position was achieved, the TSP was performed under TEE guidance. The entry into the LA was confirmed by documenting the corresponding pressure curve, and heparin was administered to maintain an ACT target of 300–400 s. Under TEE guidance, a coronary guidewire (Balance Heavy Weight, Abbott, North Chicago, NJ, USA) was carefully advanced through the inner lumen of the TSP needle into the left upper pulmonary vein (LUPV) (Figure 1D). Following that, the non-steerable sheath was advanced into the LA toward the LUPV and was subsequently replaced with a steerable TSP sheath (Vizigo™, Johnson & Johnson, New Brunswick, NJ, USA, or Agilis™, Abbott, Chicago, IL, USA) (Figure 1E). Through the steerable TSP sheath, the mapping and ablation catheters (StablePoint™, Boston Scientific, Marlborough, MA, USA; SmartTouch SF™ or Qdot™, Johnson & Johnson, New Brunswick, NJ, USA; TactiCath™ or TactiFlex™, Abbott, Chicago, IL, USA) were subsequently introduced (Figure 1F).

If PVI or a box isolation was performed, targeted remapping was carried out to confirm the isolation after the ablation. At the conclusion of the procedure, the deflected sheath was withdrawn from the left atrium (LA) into the RA and then removed from the patient. The puncture site was closed using a figure-of-eight suture. Complications such as pericardial effusion or tamponade were ruled out via TTE. Additionally, a neurological assessment was performed following cessation of sedation and upon the patient’s awakening.

In cases where the patients had an implantable cardioverter-defibrillator (ICD) or pacemaker (PM), a near-zero-fluoroscopy procedure (fluoroscopy time < 2 min) was pursued, particularly during the establishment of the LA access, to prevent lead dislocation when withdrawing the TSP system from the SVC to the RA. After achieving LA access, fluoroscopy was not used until the catheters were retracted through the RA at the end of the procedure. A single fluoroscopic image was taken in the rare cases where the esophageal probe was not visible in the 3D EAM system, although it was a 3D-mapping-integrated temperature probe.

### 2.4. Fluoroscopy Guidance Group (Fluoro Group)

The steps within the fluoroscopy guidance group (fluoro group) were fundamentally similar, though minor variations in performance were noted. Initially, the transition from the SVC to the RA and FO was monitored fluoroscopically, including two key jumps: the first from the SVC to the RA and the second into the FO. Orientation within the FO was achieved at right anterior oblique (RAO) angle of 30° for posteroanterior axis and left anterior oblique (LAO) angle of 40° for superoinferior axis, with confirmation obtained via TEE, as previously described [20]. Following the TSP, LA pressure was recorded. Similar to the zero-fluoro group, a coronary guidewire was introduced through the lumen of the TSP needle and advanced to the LUPV. Once the wire was securely positioned in the LUPV (beyond the cardiac silhouette), the TSP system was advanced into the left atrium and LUPV. Subsequently, the non-steerable transseptal sheath was replaced with a steerable one, followed by the introduction of the mapping and ablation catheter. In accordance with our established procedural workflow during this period, fluoroscopy was routinely employed as the primary guidance method. Steps such as advancing the coronary guidewire into the LUPV, exchanging the sheath, and introducing the mapping and ablation catheters were performed exclusively under fluoroscopic guidance, in contrast to the zero-fluoroscopy/near-zero-fluoroscopy workflow, where these steps were conducted under TEE guidance. TEE was routinely employed for thrombus exclusion, as well as for confirming the position of the TSP system in the region of the fossa ovalis after fluoroscopic positioning. The TEE probe was typically removed after positioning the steerable sheath in the LUPV. In cases of any abnormalities during the procedure, such as resistance or the need to confirm the position of the transseptal sheath, fluoroscopy was utilized for verification.

### 2.5. Statistics

Statistical analysis was conducted using SPSS^®^ Statistics (version 29.0.1.0, IBM, Armonk, New York, NY, USA). Compliance of continuous variables with normal distribution was assessed using the Shapiro–Wilk test. For continuous variables, measures of central tendency and variability were expressed as median and interquartile range (IQR), respectively, and comparisons were performed using the Mann–Whitney U test. Categorical variables were summarized as absolute numbers and percentages and analyzed using Chi-square or Fisher’s exact test, as appropriate. Multivariate logistic regression analysis was performed to assess whether the type of procedure influenced fluoroscopy use. A *p*-value of <0.05 was considered statistically significant.

## 3. Results

### 3.1. Baseline Characteristics

The median age of the patients was 73.0 (64.0; 79.0) years, and 58 (40.8%) were female. The cohort predominantly exhibited BMI values (27.2 (24.7; 30.4) kg/m^2^) corresponding to the categories of overweight or class I obesity. The median left ventricular ejection fraction (LVEF) was within the normal to moderately reduced range (54.0 (40.2; 60.0)%). There were no statistically significant differences observed in the cardiovascular risk factors. A detailed summary of the baseline characteristics is presented in Table 1.

### 3.2. Procedural Characteristics

A total of 53 (37.3%) patients underwent zero fluoroscopy, 20 (14.1%) underwent near-zero fluoroscopy, and 69 (48.6%) underwent fluoroscopy and TEE-guided EP. Overall, 65.0 (45.8%) patients were examined using Carto^TM^, 71 (50.0%) patients were examined using Rhythmia^TM^, and 6 (4.2%) patients were examined using EnSiteX^TM^ EAM systems. Procedural characteristics of all patients are summarized in Table 2, comparing the zero-fluoroscopy/near-zero-fluoroscopy group with the fluoroscopy group. The median procedure duration was 132.5 (107.5; 160.2) minutes, with no significant differences between the two groups (*p* = 0.52). The fluoroscopy time and dose in the zero-fluoroscopy/near-zero-fluoroscopy group were nearly 0, showing a statistically significant difference compared to the fluoroscopy group (*p* < 0.001).

Analysis of the performed procedures revealed no significant differences between the groups, with redo AF interventions being the most common (Table 3).

Multivariate analysis of fluoroscopy use, categorized into zero-fluoroscopy, near-zero-fluoroscopy, and fluoroscopy groups in relation to the performed procedure, showed no statistically significant differences. The interventions for focal left atrial tachycardias demonstrated a borderline *p*-value (*p* = 0.05; odds ratio 0.2) (Table 4).

### 3.3. Learning Curve Effects

**Figure 2:**
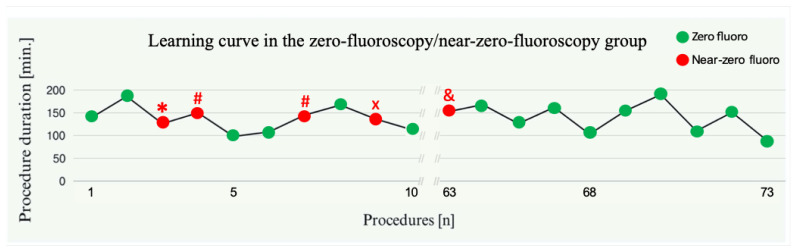
Depiction of the learning curve in the zero-fluoroscopy/near-zero-fluoroscopy group. The left side illustrates the first ten procedures, while the right side presents the last ten procedures within this group. Green dots indicate zero-fluoroscopy procedures, whereas red dots represent near-zero-fluoroscopy procedures. During the first ten procedures, fluoroscopy was utilized due to difficulties with transseptal puncture (*), the inability to visualize the esophageal temperature probe within the 3D mapping system (#), and the presence of PM/ICD leads (x). During the last ten procedures, fluoroscopy was required in only one case for pericardial drainage (&).

Throughout the learning curve in the zero-fluoroscopy/near-zero-fluoroscopy group, no significant difference in procedural duration was observed between the first and last 10 patients (*p* = 0.58) (Figure 2). Fluoroscopy was required in 40% of the initial 10 procedures, which decreased to 10% during the final 10 procedures (*p* = 0.30).

### 3.4. Reasons for Fluoroscopy Usage in the Near-Zero-Fluoroscopy Group

The reasons for fluoroscopy use in the near-zero-fluoroscopy group, consisting of 20 patients, are presented in Table 5. Fluoroscopy was required in 30% of cases for CS catheter placement in the beginning of the learning curve; in 25% of cases for visualization of the esophageal temperature probe, which could not be located with the 3D EAM system; and in 15% of cases due to PM or ICD leads.

### 3.5. Transition from Zero-Fluoroscopy to Near-Zero-Fluoroscopy Approach

In 5.5% of patients within the zero-fluoroscopy/near-zero-fluoroscopy group, a near-zero-fluoroscopy approach was planned from the beginning due to PM/ICD leads (three patients) or an ASD occluder (one patient). The remaining 94.5% were initially intended to undergo a zero-fluoroscopy procedure. However, a switch to near-zero fluoroscopy was necessary in cases of complex CS catheter placement (8.2%, six patients), an esophageal temperature probe not being visible in the 3D mapping system (6.8%, five patients), the need for pericardial drainage (4.1%, three patients), or difficult TSP (2.7%, two patients).

### 3.6. Procedural Complications

Three cases of pericardial tamponade resulting from steam pops during ablation were reported in the zero-fluoroscopy/near-zero-fluoroscopy group, whereas no such events occurred in the fluoroscopy group. This difference was not statistically significant (*p* = 0.09). No other procedure-related complications, such as left atrial perforation without steam pops, perforation during TSP or CS catheter placement, atrioesophageal fistula, or TEE-associated complications, were observed in either group.

## 4. Discussion

Over the past two decades, interventional electrophysiology has experienced remarkable growth, with catheter ablation (CA) playing a crucial role in the treatment of cardiac arrhythmias [1]. Traditionally, EP relied exclusively on fluoroscopy as the main imaging method, providing real-time guidance during interventions [21]. However, its use exposes both patients and healthcare staff to ionizing radiation, which carries inherent risks. Deterministic and stochastic effects are known concerns of radiation exposure, with even minimal doses posing cumulative risks [22]. In response to these concerns, the present study aimed to evaluate the periprocedural safety and efficacy of TEE guidance in zero-fluoroscopy/near-zero-fluoroscopy left atrial EP with 3D EAM integration.

The baseline characteristics of our patient cohort align with those reported in similar studies evaluating zero-fluoroscopy EPs [12,23,24]. Regarding procedural characteristics, there was no significant difference in the procedure duration between the zero-fluoroscopy/near-zero-fluoroscopy group and the conventional fluoroscopy group. Approximately half of the patients underwent a redo AF ablation procedure, whereas one-fifth each underwent either a first-time PVI or an ablation for left atrial AAFL. The multivariate analysis for the procedures and fluoroscopy use, categorized into zero-fluoroscopy, near-zero-fluoroscopy, and fluoroscopy groups, revealed no significant differences, except for patients with focal left atrial tachycardia, who showed borderline significance and had a fourfold lower likelihood of requiring fluoroscopy. Minimizing or completely avoiding fluoroscopy in the zero-fluoroscopy/near-zero-fluoroscopy group significantly reduces or even eliminates radiation exposure, improving safety for patients and healthcare staff in adherence to “ALARA” (as low as reasonably achievable) principles without compromising procedural efficiency [25]. The initial creation of an RA map for CS catheter placement during zero-fluoroscopy/near-zero-fluoroscopy intervention slightly extends the start of the procedure. However, the resulting RA matrix significantly improves visualization of the steerable sheath and catheter positions, enabling real-time observation of the transition through the RA into the LA and subsequently enhancing both efficiency and precision during the ablation phase.

In terms of complications, there was no statistically significant difference between the two groups. Three instances of pericardial tamponade due to steam pops during ablation occurred in the zero-fluoroscopy/near-zero-fluoroscopy group, resulting in a borderline *p*-value. Steam explosions are a well-documented phenomenon that occur during RF ablation when tissue temperatures surpass the boiling point. Ablation performed on the pulmonary vein-side of the ridge, particularly in the anterosuperior and roof segments of the LUPV, is hypothesized to increase the risk of steam pop due to greater tissue exposure. Factors such as a delta impedance exceeding 12 Ohms during ablation, high contact force, and prolonged ablation duration have been identified as potential predictors of their occurrence [26,27]. However, these parameters are not definitive, and the occurrence of steam pops remains difficult to predict. In contrast, the fluoroscopy group experienced no pericardial effusion or tamponade, a rate lower than the average complication rate of approximately 0.3–4.1% reported in other studies [28,29].

Although widely adopted worldwide, the zero-fluoroscopy/near-zero-fluoroscopy approach has its limitations. As demonstrated in our findings, fluoroscopy was required in approximately one-fifth of patients in the zero-fluoroscopy/near-zero-fluoroscopy group, despite only a small subset having a near-zero-fluoroscopy approach planned from the beginning due to the presence of intracardiac devices such as PM/ICD leads or an atrial septal defect occluder. The additional cases that were not initially anticipated in the near-zero-fluoroscopy group were most frequently due to complex CS catheter placement and an esophageal temperature probe not being visible in the 3D mapping system, followed by the less common need for pericardial drainage and difficult TSP. In all cases involving patients with PM/ICD leads, fluoroscopy was used to prevent potential lead dislocation as no adequate system currently exists to reliably monitor and confirm lead stability throughout the complete course of the leads in real time without the use of fluoroscopy. The feasibility of a completely zero-fluoroscopy approach in patients with cardiac-implantable electronic devices needs further investigation, as reported by Shimamoto et al. [30]. Fluoroscopy was also required in all patients where the esophageal temperature probe could not be reliably visualized using the 3D EAM system. A comprehensive matrix collection in the area of the LA posterior wall, however, significantly facilitated the visualization of the esophageal probe over the course of the learning curve in this study. Similarly, the use of fluoroscopy can also be avoided during the placement of CS catheters after the learning curve. This study was conducted at a center where CS catheter placement had previously been performed exclusively under fluoroscopic guidance, which had resulted in significant expertise with this technique. Consequently, during the initial implementation of the zero-fluoroscopy/near-zero-fluoroscopy approach, fluoroscopic assistance was more frequently used, particularly in cases where partial visualization of the CS catheter was difficult due to the absence of an RA matrix in certain areas. However, after the establishment of the zero-fluoroscopy/near-zero-fluoroscopy workflow, radiation was no longer required for safe and complication-free CS catheter placement.

In the learning curve analysis, there is a clear decrease in the use of avoidable fluoroscopy as a backup during the last 10 procedures compared to the first 10, although this reduction is not statistically significant. This finding aligns with our team’s experience, suggesting that a learning curve effect exists with every system to adequately visualize all catheters, including the temperature probe. This effect primarily involves gaining experience with the amount of matrix data that needs to be collected at specific locations when using the mainly magnetic-based 3D mapping systems (Carto™ and Rhythmia™). For the mainly impedance-based system (EnSiteX™), it is important to note that the TEE probe should be removed during LA procedures as its presence can otherwise cause impedance variations and visualization challenges.

Examining the learning curve associated with zero-fluoroscopy/near-zero-fluoroscopy procedures, our data indicate that procedural duration was consistent from the initial cases through to later ones. This consistency suggests that the workflow for zero-fluoroscopy/near-zero-fluoroscopy procedures is comparable to that of traditional methods, facilitating seamless integration into clinical practice without necessitating extensive additional training [31,32]. However, an additional trained TEE assistant is essential to ensure precise guidance and maintain safety throughout the procedure. Nevertheless, TEE offers significant commercial advantages over ICE, particularly given that ICE is not universally available or readily accessible as refurbished equipment. Moreover, the use of TEE obviates the need for lead aprons, thereby significantly reducing the risk of musculoskeletal injuries such as herniated discs [33,34] among healthcare staff, including medical technical assistants. This improvement contributes to enhanced ergonomics and greater procedural comfort. Finally, it is important to emphasize that the zero-fluoroscopy/near-zero-fluoroscopy approach has been seamlessly integrated into our routine clinical practice, demonstrating its feasibility and reliability as a standard procedural technique.

### Limitations

This study has several limitations. The small cohort size may affect the generalizability of the findings. Its prospective, non-randomized design introduces potential selection bias. Additionally, the analysis is limited to periprocedural outcomes, without long-term follow-up to assess the durability and safety of the approach.

## 5. Conclusions

This study demonstrates the feasibility and safety of using TEE guidance in zero-fluoroscopy/near-zero-fluoroscopy left atrial EP. This approach significantly reduces or eliminates radiation exposure without prolonging procedure times or increasing complication rates. While the majority of EPs can already be performed without radiation using 3D mapping systems combined with TEE, certain anatomical complexities and device-related factors still necessitate fluoroscopy. Further large-scale randomized studies are needed to better assess the reliability and limitations of TEE guidance alone in EPs.

## Figures and Tables

**Table 1:** Baseline characteristics of the patients in the zero-fluoroscopy/near-zero-fluoroscopy and fluoroscopy groups.

Baseline Characteristics	All Patients (*n* = 142)	Zero-Fluoro/Near-Zero-Fluoro Group (*n* = 73)	Fluoro Group (*n* = 69)	*p*-Value
Age [years], median (IQR)	73.0 (64.0; 79.0)	70.5 (62.2; 77.7)	72.0 (67.0; 79.5)	0.05
Female, *n* (%)	58.0 (40.8)	29.0 (39.7)	29.0 (42.0)	0.78
BMI [kg/m^2^], median (IQR)	27.2 (24.7; 30.4)	27.4 (25.0; 29.7)	26.9 (24.7; 30.6)	0.68
CHA_2_DS_2_-VA score, median (IQR)	3.0 (2.0; 4.0)	3.0 (2.0; 4.0)	3.0 (2.0; 4.0)	0.46
LVEF (%), median (IQR)	54.0 (40.2; 60.0)	56.0 (41.7; 60.7)	50.5 (38.5; 60.0)	0.36
LA diameter [cm], median (IQR)	4.4 (3.7; 5.2)	4.2 (3.5; 5.2)	4.6 (3.9; 5.3)	0.20
Arterial hypertension, *n* (%)	100.0 (70.4)	53.0 (72.6)	47.0 (68.1)	0.56
Diabetes mellitus, *n* (%)	23.0 (16.2)	13.0 (17.8)	10.0 (14.5)	0.59
Coronary artery disease, *n* (%)	61.0 (43.0)	29.0 (39.7)	32.0 (46.4)	0.42
OSA, *n* (%)	16.0 (11.3)	7.0 (9.6)	9.0 (13.0)	0.51
COPD, *n* (%)	6.0 (4.2)	1.0 (1.4)	5.0 (7.2)	0.08
Positive family history for vascular or cardiac events, *n* (%)	21.0 (14.8)	10.0 (13.7)	11.0 (15.9)	0.71
Former or current nicotine abuse, *n* (%)	35.0 (24.6)	15.0 (20.5)	20.0 (29.0)	0.44

BMI, body mass index; COPD, chronic obstructive pulmonary disease; IQR, interquartile range; LA, left atrium; LVEF, left ventricular ejection fraction; OSA, obstructive sleep apnea.

**Table 2:** Procedural characteristics of the patients in the zero-fluoroscopy, near-zero-fluoroscopy, combined zero-fluoroscopy/near-zero-fluoroscopy, and fluoroscopy groups.

Procedural Characteristics	All Patients (*n* = 142)	Zero-Fluoro Group (*n* = 53)	Near-Zero-Fluoro Group (*n* = 20)	Combined Zero-Fluoro/Near-Zero-Fluoro Group (*n* = 73)	Fluoro Group (*n* = 69)	*p*-Value
Procedure duration [minutes], median (IQR)	132.5 (107.5; 160.2)	130.0 (107.0; 157.0)	140.5 (127.0; 165.5)	132.0 (110.0; 157.0)	133.0 (101.5; 161.5)	0.52
Fluoroscopy time [minutes], median (IQR)	2.3 (0.0; 9.6)	0	0.4 (0; 1.4)	0	9.7 (5.9; 15.3)	<0.001
Fluoroscopy dose [cGy·cm^2^], median (IQR)	95.4 (0.0; 740.1)	0	16.9 (5.5; 51.8)	0.0 (0.0; 0.3)	755.8 (519.0; 1467.9)	<0.001

IQR, interquartile range.

**Table 3:** Procedures performed in the zero-fluoroscopy, near-zero-fluoroscopy, combined zero-fluoroscopy/near-zero-fluoroscopy, and fluoroscopy groups.

Procedures	All Patients (*n* = 142)	Zero-Fluoro Group (*n* = 53)	Near-Zero-Fluoro Group (*n* = 20)	Combined Zero-Fluoro/Near-Zero-Fluoro Group (*n* = 73)	Fluoro Group (*n* = 69)	*p*-Value
First-time PVI, *n* (%)	31.0 (21.8)	7.0 (13.2)	4.0 (20.0)	11.0 (15.1)	20.0 (29.0)	0.07
Redo AF ablation, *n* (%)	69.0 (48.6)	24.0 (45.3)	9.0 (45.0)	33.0 (45.2)	36.0 (52.2)	0.50
Atypical LA AFL, *n* (%)	27.0 (19.0)	13.0 (24.5)	3.0 (15.0)	16.0 (21.9)	11.0 (15.9)	0.49
Focal LAT, *n* (%)	11.0 (7.7)	7.0 (13.2)	2.0 (10.0)	9.0 (12.3)	2.0 (2.9)	0.07
WPW syndrome, *n* (%)	4.0 (2.8)	2.0 (3.8)	2.0 (10.0)	4.0 (5.5)	0	0.12

AF, atrial fibrillation; AFL, atrial flutter; LA, left atrial; LAT, left atrial tachycardia; PVI, pulmonary vein isolation; WPW, Wolff–Parkinson–White.

**Table 4:** Multivariate analysis of fluoroscopy use, categorized into zero-fluoroscopy, near-zero-fluoroscopy, and fluoroscopy groups in relation to the performed procedure.

	Regression Coefficient	Standard Error	z-Value	Sig.	Odds Ratio	95% Confidence Interval
First-time PVI, *n* (%)	0.5	0.4	1.1	0.25	1.7	0.7–4.0
Redo AF ablation, *n* (%)	−0.5	0.4	1.1	0.25	0.6	0.2–1.4
Atypical LA AFL, *n* (%)	−0.5	0.5	1	0.31	0.6	0.3–1.5
Focal LAT, *n* (%)	−1.6	0.8	1.9	0.05	0.2	0.1–1.0
WPW syndrome, *n* (%)	−20.4	7968.5	0	0.99	N/A *	N/A *

* Not applicable due to an insufficient sample size. AF, atrial fibrillation; AFL, atrial flutter; LA, left atrial; LAT, left atrial tachycardia; N/A, not applicable; PVI, pulmonary vein isolation; Sig., significance; WPW, Wolff–Parkinson–White.

**Table 5:** Reasons for fluoroscopy usage in the near-zero-fluoroscopy group.

Reasons for Fluoroscopy Usage	Near-Zero-Fluoroscopy Group (*n* = 20)
Complex CS catheter placement, *n* (%)	6.0 (30.0)
Esophageal temperature probe not visible, *n* (%)	5.0 (25.0)
PM/ICD leads, *n* (%)	3.0 (15.0)
Pericardial drainage, *n* (%)	3.0 (15.0)
Difficult TSP, *n* (%)	2.0 (10.0)
ASD occluder, *n* (%)	1.0 (5.0)

ASD, atrial septum defect; CS, coronary sinus; ICD, implantable cardioverter-defibrillator; PM, pacemaker; TSP, transseptal puncture.

## Data Availability

The data presented in this study are available on request from the authors. The data are not publicly available due to data privacy laws.
